# High Throughput Method to Quantify Anterior-Posterior Polarity of T-Cells and Epithelial Cells

**DOI:** 10.3390/v3122396

**Published:** 2011-11-28

**Authors:** Charletha V. Irvin-Wilson, Justin Y. Newberg, Kathleen Kong, Ronald T. Javier, Susan J. Marriott

**Affiliations:** 1 Department of Molecular Virology and Microbiology, Baylor College of Medicine, One Baylor Plaza, Houston, TX 77030, USA; Email: irvinwil@bcm.edu (C.V.I.-W.); kong@bcm.edu (K.K.); rjavier@bcm.edu (R.T.J.); 2 Department of Molecular and Cellular Biology, Baylor College of Medicine, One Baylor Plaza, Houston, TX 77030, USA; Email: newberg@bcm.edu

**Keywords:** polarity, high throughput imaging, immunological synapse, virological synapse, T-cell, epithelial cell, pipeline pilot

## Abstract

The virologic synapse (VS), which is formed between a virus-infected and uninfected cell, plays a central role in the transmission of certain viruses, such as HIV and HTLV-1. During VS formation, HTLV-1-infected T-cells polarize cellular and viral proteins toward the uninfected T-cell. This polarization resembles anterior-posterior cell polarity induced by immunological synapse (IS) formation, which is more extensively characterized than VS formation and occurs when a T-cell interacts with an antigen-presenting cell. One measure of cell polarity induced by both IS or VS formation is the repositioning of the microtubule organizing center (MTOC) relative to the contact point with the interacting cell. Here we describe an automated, high throughput system to score repositioning of the MTOC and thereby cell polarity establishment. The method rapidly and accurately calculates the angle between the MTOC and the IS for thousands of cells. We also show that the system can be adapted to score anterior-posterior polarity establishment of epithelial cells. This general approach represents a significant advancement over manual cell polarity scoring, which is subject to experimenter bias and requires more time and effort to evaluate large numbers of cells.

## 1. Introduction

Anterior-posterior (AP) cell polarity is defined as the asymmetric distribution of macromolecules and organelles in the x-y cell plane. This process, which becomes initiated by localized activation of receptors at the cell surface, is essential for diverse functions, including embryonic morphogenesis, tissue repair, and immune surveillance [1,2,3,4]. AP cell polarity is also exploited by HIV and HTLV-1 to form the virological synapse (VS), where virus cell-cell spread takes place [5,6]. Normal AP cell polarity is also disrupted by viruses [6,7,8,9]. For example, Barnard *et al.* showed that in T-cells, HTLV‑1 blocks normal AP cell polarization while promoting AP cell polarization associated with VS formation [10].

The immunological synapse (IS) is a specialized cell-cell contact membrane junction formed by the interaction between a T-cell and antigen-presenting cell. Formation of the IS triggers activation of cell signaling cascades that promote AP polarization and contribute to activation, survival, and migration of T-cells [6,11,12]. During the process of AP polarization in T-cells, the microtubule-organizing center (MTOC) changes from a random orientation to an orientation near the IS of the cell and therefore represents a useful marker to score AP cell polarity [5,12,13].

Previous studies have scored AP cell polarity establishment in microscopic images using a time consuming and laborious manual process [5,10,14,15,16,17,18]. To improve upon this method, we have developed a new automated, high throughput technique to score AP polarity establishment in both T‑cells and epithelial cells. This automated system should facilitate all studies of AP polarity, as well as those directed at investigating how viruses perturb and exploit this important cellular process.

## 2. Results and Discussion

### 2.1. Results

**Figure 1:**
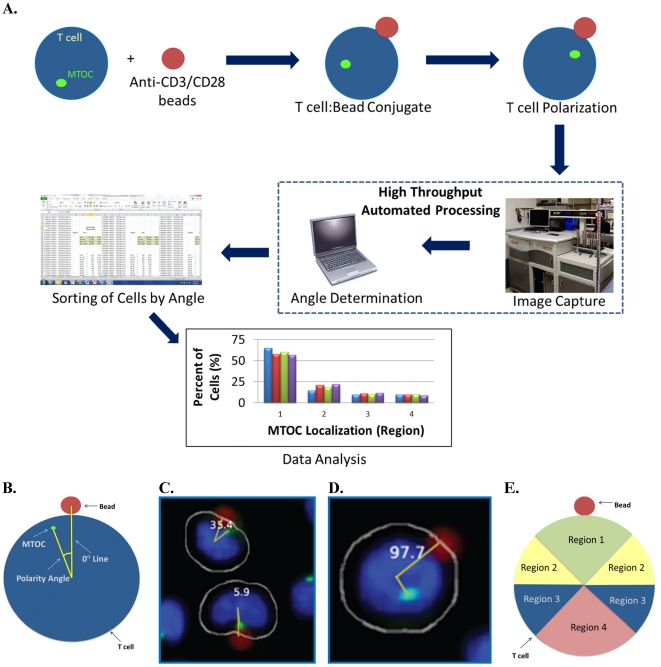
Automated MTOC:bead angle determinations. (**A**) Outline of overall experimental process including cell:bead conjugation, automated high throughput imaging and MTOC:bead angle calculation, and data analyses. (**B**) Definitions of the 0° and MTOC lines. The smallest angle formed between these two lines defines the MTOC:bead angle. (**C**/**D**) Example images showing cells with different MTOC:bead angles. The computer generated cell mask (white outline) that simulates the cell perimeter is also shown. (**E**) The four 90° cell regions (1–4) into which MTOC:bead angles for cell populations are sorted are shown. Skewed cell distributions in these regions indicate cell polarity establishment.

To induce IS formation and AP polarity establishment, we chose the established method of incubating human Jurkat T-cells with anti-CD3/CD28 coated beads [5]. Microscopic imaging of cell:bead conjugates requires attachment to a slide or plate. While centrifugation of non-adherent T cells is often used for this purpose [5], this method causes Jurkat cells to have an abnormal morphology and irregular size (data not shown), which interferes with automated analyses detailed below. This problem is circumvented by allowing cell:bead conjugates to settle by gravity onto plates, thereby retaining normal Jurkat cell morphology. Binding of the anti‑CD3/CD28 bead to the Jurkat cell subsequently induces AP polarity establishment characterized by redistribution of the MTOC near the IS formed at the bead-cell contact point. Thus, to assess cell polarity establishment, we must visualize the MOTC, which is stained with anti-pericentrin antibody [19]. DAPI staining additionally permits visualization of nuclei, whereas beads are detected directly due to their red color. The automated high throughput system then captures microscopic images of cell fields and uses the relative locations of the MTOC, nucleus, and bead of each cell:bead conjugate to calculate a MTOC:bead angle. Finally, MTOC:bead angles output from the automated system are analyzed to score T-cell polarity establishment for a cell population. The overall procedure is outlined in Figure 1A.

In the first step of the automated system, confocal microscopy images are collected and compiled by a Pipeline Pilot algorithm. The algorithm determines the eligibility of each cell for MTOC:bead angle determination based on two strict criteria. (1) The cell must contact only one bead and must not contact another cell to ensure that the cell receives only one polarization signal. (2) The cell must contain only one MTOC. Cells meeting both criteria are subjected to the second step of the automated system, in which a reference line, designated the 0° line, is drawn between the center of the nucleus and the center of the bead attached to this cell (Figure 1B). The system also draws a second line, designated the MTOC line, between the center of the nucleus and center of the MTOC. In each cell, the 0° and MTOC lines intersect at the center of the nucleus to define the MTOC:bead angle, which is the smallest angle formed between the two lines and which is accurate to four significant digits, with the dimension of a pixel representing the limiting factor. The Pipeline Pilot algorithm additionally allows one to view compiled images showing the MTOC:bead angle for each cell (Figures 1C,D). Images include the white cell mask, green-stained MTOC, blue-stained nucleus, and red-colored bead. The algorithm creates the cell mask to simulate the cell perimeter based on information obtained about the unique size and shape of Jurkat cells.

To assess cell polarity establishment, MTOC:bead angles calculated for cell sample populations are compiled and sorted into four different 90° regions: region 1 includes two adjacent 45° sectors bisected by the 0° line; region 2 includes two separate 45° sectors adjacent to and on either side of region 1; region 3 includes two separate 45° sectors adjacent to and on either side of region 2; region 4 includes two adjacent 45° sectors next to region 3 (Figure 1E). Thus, by definition, region 1 is closest to the bead whereas regions with higher numbers are progressively further from the bead. Sorted data are then subjected to an ANOVA analysis to determine whether the number of cells found in any region is statistically different from that of other regions. Theoretically, unpolarized cells should show equal (25%) distribution within regions 1–4, whereas polarized cells should show higher distribution within region 1 and concomitantly lower equivalent distributions in regions 2–4.

**Figure 2:**
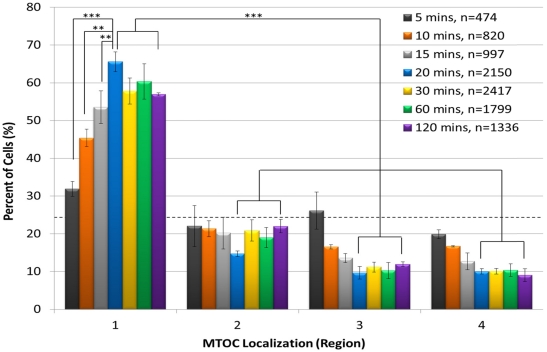
Anti-CD3/CD28 beads rapidly induce T-cell polarization. Jurkat T cells were incubated with anti-CD3/CD28 beads for 5, 10, 15, 20, 30, 60, or 120 min, stained with DAPI and for pericentrin, and subjected to automated imaging and MTOC:bead angle determinations. The percentage of each cell sample population that sorted into regions 1–4 is shown. The number of scored cells (n) is shown in the legend for each time point. The horizontal dashed line indicates the 25% random cell distribution within each region expected for unpolarized cells. **, p < 0.007; ***, p < 0.001.

We first used the automated system to establish the kinetics of AP polarity establishment following incubation of Jurkat cells with anti-CD3/CD28 beads for 5, 10, 15, 20, 30, 60 or 120 min (Figure 2). The results showed that the percentage of cells containing an MTOC in region 1 progressively increased from 0 min, peaked at 20 min, and remained approximately steady thereafter through the 120 min time point. As expected, we also observed that a smaller percentage of cells contained the MTOC in regions 2–4 than in region 1 at all time points (Figure 2). Differences between the numbers of cells containing an MTOC in region 1 and those with an MTOC in regions 2–4 were statistically significant for the 20, 30, 60 and 120 min time points. These findings show that T-cell polarization occurs rapidly, as it is detected as early as 5 min after addition of anti-CD3/CD28 beads. Also notable was that the automated system permitted imaging of large numbers of cells fitting our strict scoring criteria at each time point. For example, the lowest number of scored cells was 474 at the 5 min time point, presumably due to the short time available for cells to adhere to the plate surface and to attach to beads. In contrast, the number of scored cells increased to a maximum of 2,417 at 30 min of conjugation time. We considered scored cell numbers greater than 1,000 to be optimal for statistical analysis. Because the 20 min time point yielded the peak level of cell polarization and >1000 scored cells, it was used in all subsequent polarity experiments.

**Figure 3:**
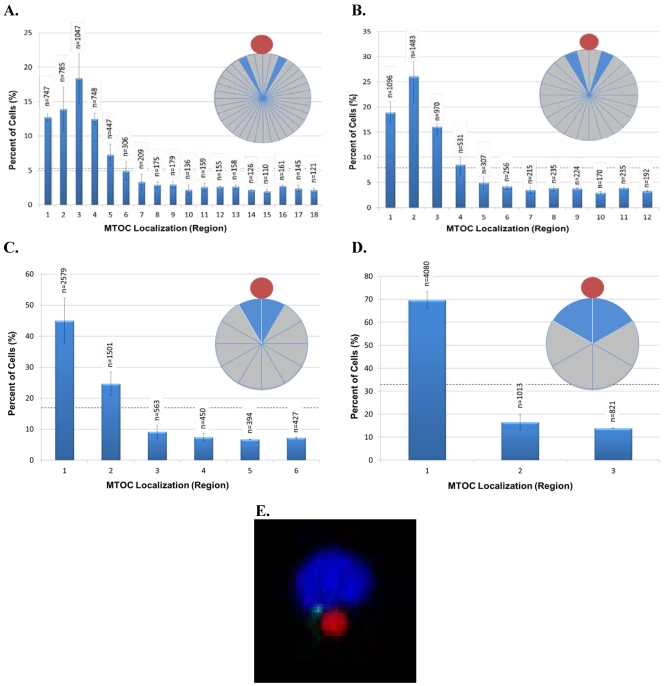
High resolution analysis of MTOC position during T-cell polarization. Data from the 20 min time point in Figure 2 and from an independent replicate were analyzed and re‑sorted into regions of 20° (**A**), 30° (**B**), 60° (**C**), and 120° (**D**). As defined in Figure 1E, region 1 is always closest to the bead, whereas regions with higher numbers are progressively farther from the bead. The inset pie diagrams in each panel illustrate the regions analyzed, with the blue region indicating the most frequent MTOC localization in the cell population. The number of scored cells (n) in each region is shown above each bar. The black horizontal dashed line indicates the random cell distribution expected within each region for unpolarized cells. Error bars represent the standard error of the mean of two independent experiments. (**E**) Example of the bead-induced cell dimple postulated to restrict MTOC movement into region 1 and MTOC positioning on the 0° line, as seen in Figures 3A,B.

We next investigated whether grouping cells in smaller or larger sized regions would yield additional information about cell polarity. For this purpose, we sorted data from a new 20 min time point experiment, as well as re-sorted Figure 2 data for the 20 min time point into eighteen 20° regions, twelve 30° regions, six 60° regions, or three 120° regions and then graphed the results (Figures 3A–D). For all of these analyses, we expected that the highest percentage of cells would have their MTOC located within region 1, which is closest to the bead. This was observed in the 60° and 120° regions (Figures 3C,D). However, an interesting and unexpected result was obtained from the analyses of the higher resolution 20° and 30° regions. For 20° regions, the highest percentage of cells instead had their MTOC located within region 3 (20°–30° from 0° line) (Figure 3A) and, for 30° regions, the highest percentage of cells instead had their MTOC located within region 2 (15°–30° from 0° line) (Figure 3B). The same result was seen for 20° and 30° region analyses conducted with data from the 30, 60, and 120 min time points of Figure 2 (data not shown). Because the anti-CD3/CD28 bead frequently caused an indentation, or dimple, in the Jurkat cell at the bead:cell contact point (Figure 3E), it is possible that the dimple creates a physical barrier that reduces MTOC movement into region 1 and MTOC positioning on the 0° line in Figures 3A,B. The results in Figure 3A additionally revealed that cell polarity establishment manifests as MTOC accumulation within a 100° sector bisected by the 0° line (regions 1–5), thereby validating our original choice of using four 90° regions to evaluate AP polarity establishment in Jurkat cells (Figure 1E). More important, our ability to discern high resolution MTOC movement in as small as 20° cell regions underscores the power of this automated, high throughput method to provide new insights into cell polarity establishment.

**Figure 4:**
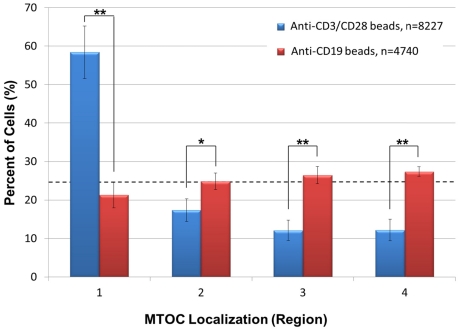
Anti-CD19 beads fail to promote T-cell polarization. Jurkat cells were incubated with negative control anti-CD19 beads or positive control anti-CD3/CD28 beads for 20 min, and stained with anti-pericentrin antibody and DAPI. High throughput image acquisition and angle analysis was performed. The percentage of cells containing an MTOC in the indicated region is shown. The graph represents three independent experiments. The number of scored cells (n) is shown in legend. The horizontal dashed line indicates the 25% random cell distribution within each region expected for unpolarized cells. *, p < 0.05; **, p < 0.002.

As a control, we also asked whether anti-CD3/CD28 beads specifically promote polarity establishment in Jurkat T-cells. This was accomplished by incubating Jurkat cells for 20 min with negative control anti-CD19 coated beads, which do not bind to or elicit IS formation or polarity establishment in Jurkat cells [20], and then by subjecting the cells to the same automated high throughput analysis described in Figure 2. The results indicated that anti-CD19 beads fail to promote Jurkat cell polarization as evidenced by approximately equivalent numbers of cells sorting into regions 1–4 (Figure 4). In the same experiment, however, positive control Jurkat cells incubated with anti-CD3/CD28 beads showed a statistically significantly higher number of cells containing an MTOC in region 1 than in regions 2–4, demonstrating cell polarity establishment (Figure 4). Previous work showing that anti‑CD58 beads bind to Jurkat T-cells but fail to induce anterior-posterior cell polarization [10] indicates that formation of bead:cell conjugates is not sufficient to induce cell polarization, further illustrating the specificity of our results with anti-CD3/CD28 beads.

**Figure 5:**
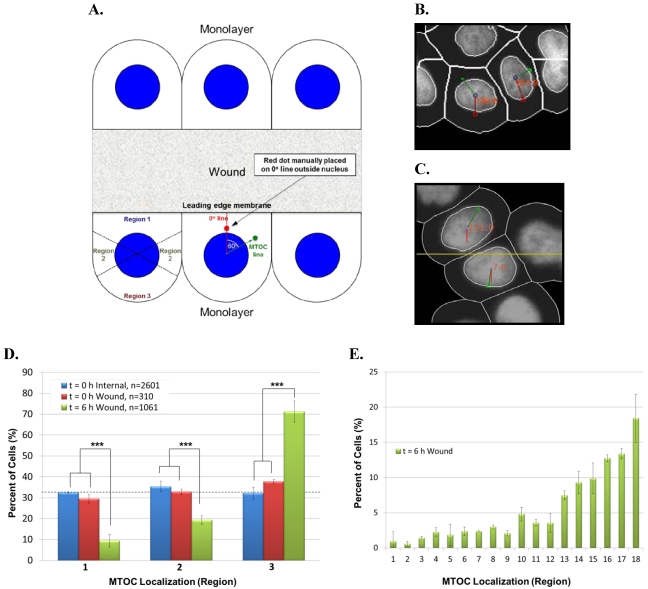
Use of the automated system to score AP polarity establishment in human MCF‑10A mammary epithelial cells. (**A**) Illustration of method used to calculate MTOC:leading edge angles in wound-edge cells. In images of wound-edge cells stained with anti-pericentrin antibody and DAPI, the automated system uses the MTOC (green dot), manually placed red dot, and center of the nucleus to draw the 0° and MTOC lines and to calculate the smallest MTOC:leading edge angle, as detailed in the text. The illustrated example shows an MTOC:leading edge angle of 60° (lower middle cell). MTOC:leading edge angles are used to sort the cell population into regions 1–3 to evaluate cell polarity establishment (lower left cell). (**B**) Image of cells showing MTOC:leading edge membrane angles calculated by the automated system. (**C**) Method for calculating MTOC:artificial leading edge angle in internal monolayer cells. In images of unwounded monolayer cells stained for pericentrin, the automated system uses the MTOC (green dot), the artificial wound (yellow line), and center of the nucleus to draw the 0° line (red) and the MTOC line (green line) and to calculate the smallest MTOC:artificial leading edge angle, as detailed in the text. (**D**) Bar graph showing the percentage of the indicated cell samples sorting into regions 1–3. Bars represent mean and standard deviation of three sets of each group of cells. Total number of cells (n) in each group is indicated. The horizontal dashed line indicates the random cell distribution within each region expected for unpolarized cells. ***, p < 0.001 for corresponding regions of both t = 0 h control samples. (**E**) Data from the 6 h time point in Figure 5D was re-sorted into 20° regions. Bars represent mean and standard deviation of three sets of the t = 6 h wound-edge cells.

To determine whether our high throughput imaging system could be applied to systems other than Jurkat T-cells, we next adapted the automated angle calculation system to score AP polarity establishment in epithelial cells. In human MCF-10A mammary epithelial cells, cell surface receptors initiate signaling cascades in response to a polarizing stimulus, such as wounding of a cell monolayer. In wound-edge cells, this stimulus promotes forward microtubule polarization toward the wound and rearward reorientation of the Golgi apparatus and MTOC behind the nucleus. Thus, as for Jurkat cells, we used the MTOC as a marker to score polarity establishment, which was determined by the MTOC distribution within three 120° regions (regions 1–3) of wound-edge MCF-10A cells (Figure 5A).

To test the automated system on MCF-10A cells, we overnight EGF-starved a confluent cell monolayer, which was then scratch-wounded multiple times with a P200 pipette tip. Negative control cells were fixed immediately post-wounding (t = 0 h), whereas experimental cells were incubated in complete medium for 6 h post wounding to allow for cell polarization and then fixed. Cells were stained with anti-pericentrin antibody and DAPI, and images of cell fields were collected manually. In cell images, Adobe Photoshop was used visually to place a red dot in the cytoplasm between the center of the nucleus and the center of leading edge membrane. The automated system was employed (1) to connect the center of the red dot to the center of the nucleus, thereby creating the reference 0° line, (2) to connect the center of the MTOC to the center of the nucleus, thereby creating the MTOC line, and (3) to calculate the smallest MTOC:leading edge angle formed by the intersection of the 0° and MTOC lines (Figure 5A). Images of actual cells scored by the automated system are shown in Figure 5B.

As an additional negative control for cell polarity establishment, we similarly scored images of internal monolayer cells stained for pericentrin. In this case, however, an artificial wound was introduced by using Adobe Photoshop to draw a straight arbitrary line (yellow) onto images of unwounded cell monolayers. The automated scoring system was then used to calculate the smallest angle formed between the 0° line (red), which is perpendicular to the artificial wound edge and runs through the center of nucleus, and the MTOC line (green), which connects the center of the MTOC to the center of the nucleus (Figure 5C). Theoretically, both t = 0 h wound-edge cells and internal monolayer cells should score as unpolarized (MTOC randomly distributed around the nucleus) as evidenced by approximately 33.3% of the cells sorting into regions 1–3. In contrast, t = 6 h wound‑edge cells should have a significantly higher percentage of cells sorting into region 3 than into either region 1 or region 2.

Results in Figure 5D show that the percentage of negative control t = 0 h wound-edge cells and internal monolayer cells sorting into regions 1–3 was comparable, approximating the theoretical value of 33.3% expected for unpolarized cells. By contrast, 71% of t = 6 h wound-edge cells sorted into region 3, similar to 70–80% reported previously [21], whereas only 9% or 19% of cells sorted into regions 1 and 2, respectively. Moreover, the numbers of t = 6 h wound-edge cells are statistically significantly different than those of t = 0 h wound-edge cells and internal monolayer cells in all corresponding regions, demonstrating cell polarity establishment.

As was done with Jurkat T-cells in Figure 3E, we re-sorted data from the 6 h time point in Figure 5D into 20° regions. The results showed that the highest percentage of polarized MCF-10A cells had their MTOC located in region 18, a cell sector diametrically opposed to the wound edge (Figure 5E). This finding reveals that the MTOC distribution in polarized MCF‑10A cells peaks on the cell-bisecting line that passes through the centers of the leading edge membrane and nucleus. This differs from polarized Jurkat T‑cells, where the MTOC distribution instead peaks at angles slightly offset from an equivalent cell-bisecting line (see Figure 3A). Results in Figure 5E additionally validated the use of 120° regions to evaluate AP polarity establishment in MCF-10A cells as their polarization manifested as MTOC accumulation within regions 13–18.

### 2.2. Discussion

We described the development of a new automated, high throughput method to score AP cell polarity establishment by capturing cell images while excluding abnormal cells using a strict set of criteria, calculating precise MTOC:bead or MTOC:leading edge angles, and compiling the data. In addition, flexibility of the approach allowed us to analyze cells of different types and to sort them into cell regions of various sizes. The system also facilitated data collection for thousands of cells. These key features permit detection of statistically significant differences even between small cell regions, providing high-resolution insights into the mechanisms of cell polarity establishment.

The versatility of our algorithm also allowed us to test a detailed hypothesis. We initially sorted Jurkat T‑cell populations into four 90° regions. In this case, unpolarized cells should distribute approximately equally (25% of cells/region) within each region, whereas polarized cells should instead distribute predominantly in the region encompassing the polarization stimulus (anti-CD3/CD28 coated bead contact site). Indeed, in Jurkat cells, we observed a significantly increased accumulation of MTOC in regions adjacent to the site of bead contact, an effect that was maximal after a 20 min incubation period. This finding led us to hypothesize that the MTOC migrates relatively rapidly to a position immediately adjacent to the cell polarization stimulus. We investigated this idea by re-sorting cells into smaller 20° or 30° regions. To our surprise, we found that the MTOC does not concentrate closest to the bead contact site, but rather to a 20–30° region on either side of this site (region 3 in Figure 3A or region 2 in Figure 3B). This finding led us to observe that many cells become dimpled at the point of bead contact, and to suspect that this altered cell architecture may restrict MTOC movement into this region. Also possible is that cellular factors or pathways restrict MTOC migration into this region or anchor the MTOC at a position offset from this region. Determining the molecular basis for this effect requires additional study.

To determine whether the automated system could be adapted to other cell types, we modified the algorithm to score polarity in adherent MCF-10A epithelial cells. At present, a reference point is manually added to the images of epithelial cells. To fully automate polarity scoring of migrating epithelial cells, we plan in the future to use an antibody to mark the leading edge membrane of wound edge cells and to develop an algorithm that places a dot at the center of the leading edge membrane. We succeeded in scoring polarity from large numbers of epithelial cells. Furthermore, contrary to polarized Jurkat T cells, the MTOC of polarized MCF-10A cells is known to concentrate in the region directly opposite from the polarizing stimulus. Indeed, we observed maximal MTOC accumulation in the region directly opposite from the polarizing stimulus, rather than offset from the predicted pattern seen in Jurkat cells. The difference between MTOC accumulation patterns in Jurkat and MCF-10A cells may depend on the physical nature of the stimulus itself as no equivalent to the bead-induced dimple in Jurkat T cells is seen in scratch-wounded MCF‑10A epithelial cells. It is also possible that MTOC migration away from the polarizing stimulus in MCF-10A cells does not enforce the same physical restriction for MTOC movement postulated in Jurkat cells.

The new automated system also has important virological applications. The loss of cell polarity is a feature of epithelial-derived cancer cells, and there is increasing evidence that this defect in polarity may play a pivotal role in the pathogenicity of cancer [22]. In support of this idea, several oncogenic human viruses are known to target and affect cell polarity proteins [22,23,24,25]. For example the E4-ORF1 oncoprotein from human adenovirus (Ad) type 9 binds cell polarity proteins Dlg1, PATJ, and ZO-2 [22]; the E6 oncoprotein of high-risk human papillomavirus (HPV) binds cell polarity proteins Dlg1, PATJ, and Scribble [22,24]; and the Tax oncoprotein of human T-cell leukemia virus type 1 (HTLV-1) binds cell polarity proteins Dlg1 and Scribble [26]. Thus, our original goal was to develop an assay to facilitate scoring of polarity in T‑cells and epithelial cells, the natural host cells of HTLV-1, Ad, and HPV. We propose that this automated system can be used to provide key mechanistic insights about how cells polarize and how viruses disturb this process to alter cell growth and spread cell to cell. In summary, the high resolution use of this flexible, high throughput system will likely help to reveal new biologic mechanisms.

## 3. Experimental Section

### 3.1. Cells

Human Jurkat T cells and MCF-10A human mammary epithelial cells were acquired from the American Type Culture Collection (Manassas, VA, USA). Jurkat cells were maintained in RPMI 1640 media with L-glutamine containing 10% fetal bovine serum (FBS) and antibiotic-antimycotic solution (1,000 units penicillin, 1,000 µg streptomycin, and 0.25 µg amphotericin B per mL), whereas MCF‑10A human mammary epithelial cells were maintained in Dulbecco’s Modified Eagle Medium:F12 supplemented with 5% horse serum, 10 µg/mL insulin, 0.5 µg/mL hydrocortisone, 100 ng/mL cholera toxin, 20 ng/mL EGF, and 20 µg/mL gentamicin. All cells were cultured at 37 °C under a 5% CO_2_ atmosphere.

### 3.2. Jurkat T-Cell IS Formation and Immunofluorescence Assays

Exponentially growing Jurkat cells and Dynabeads conjugated to anti-CD3/CD28 (Invitrogen Dynal AS, Oslo, Norway, catalog # 111.31D) or anti-CD19 (catalog # 111.43D) antibodies were separately washed in PBS (Invitrogen catalog # 14190-144) containing 0.5% FBS (PBS FBS buffer). Washed Jurkat cells (8 × 10^4^) mixed with beads (4 × 10^4^) in 0.1 ml total volume of PBS FBS buffer were added to each poly-L-lysine-coated well of a 96-well optical grade plate (Greiner Bio-One, Monroe, North Carolina, catalog # 655892P) and allowed to settle for 20 min at 37 °C in a tissue culture incubator. After aspiration of the PBS-FBS buffer, cells were fixed in 3.5% paraformaldehyde (PFA) in PBS for 10 min at room temperature (RT), rinsed, and permeabilized with 0.1% Triton-X 100 in PEM buffer for 30 min at RT. Permeabilized cells were washed three times for 5 min, blocked in filtered IF buffer containing 10% goat serum for 1 h at RT, and incubated with rabbit anti-pericentrin (1:1000) (Abcam, Cambridge, MA, catalog # ab4448) in KB buffer for 1 h at 37 °C in a tissue culture incubator. After incubation with the primary antibody, cells were washed three times for 5 min, incubated with goat anti-rabbit AlexaFluor-488 (1:5000) (Invitrogen, Carlsbad, California catalog # A-11008) in KB buffer for 45 min at 37 °C in a tissue culture incubator, washed three times for 5 min, counterstained with 1 µg/µL DAPI, and rinsed with PBS. Unless otherwise indicated, rinses and washes mentioned above were done with PEM buffer (80 mM PIPES, pH 6.8, 5mM EGTA, pH 7.0, and 2 mM MgCl_2_). Images were collected on a Beckman IC100 high throughput microscope at the Baylor College of Medicine Integrated Microscopy Core.

### 3.3. Epithelial Cell Scratch Wound Assays and Immunofluorescence Assays

MCF-10A cells were seeded at 3 × 10^5^ cells/well on 2-well glass slides in complete medium and, upon reaching confluency, were incubated overnight in complete medium lacking EGF. The cell monolayer was then scratch-wounded five times per well using a P200 pipette tip, washed with complete medium lacking EGF to remove detached cells, and incubated in complete media for 6 h. Immunofluorescence assays were conducted as described for Jurkat cells, except PFA was quenched with 7.5% glycine solution prior to blocking with goat serum, goat anti-rabbit AlexaFluor-488 (1:500) (Invitrogen, Carlsbad, California catalog # A-11008) secondary antibody was used, and images were captured on a Zeiss Axioplan upright microscope or a Nikon A1 confocal microscope at the Baylor College of Medicine Integrated Microscopy Core.

### 3.4. High Throughput Image Collection, Preprocessing, Segmentation, Filtering, and Angle Measurement Epithelial Cell Scratch Wound Assays and Immunofluorescence Assays

Background subtracted from images was approximated as m + m^1/2^, where m is the minimum pixel intensity of the image. Objects (sets of contiguous foreground pixels) in different channels were detected using a local threshold approach in which an image pixel is placed in the foreground if its intensity is greater than the mean intensity in a surrounding window plus some constant, C, which was empirically assigned based on its ability to exclude non-objects and retain real objects. Holes in objects were filled to make them solid. Any objects touching the border of an image field were excluded. Nuclei were detected in the DAPI channel using a window of 11 × 11 pixels and C = 3; MTOC were detected in the GFP channel using a window of 7 × 7 pixels and C = 10; and beads were detected in the red channel using a window of 7 × 7 pixels and C = 20.

Cell boundaries were approximated from the nuclear objects using a distance map, whose pixels have values based on their distance in pixels to the nearest nuclear foreground pixel. Contiguous pixels in the distance map with a value of five or less were used to define cell objects. Beads that did not overlap with cells were excluded. Next, the centers of all objects in an image field were calculated, and these measurements were used to associate a cell with the closest bead and MTOC. Cells associated with more than one bead were also excluded. For the remaining cells, the MTOC:bead angle, defined as the smallest angle between a line from the center of the nucleus to the center of the bead (0° reference line) and a line from the center of the nucleus to the center of the MTOC (MTOC line), was calculated. Automated image preprocessing, segmentation, filtering, and measurement were performed in Pipeline Pilot 8.0 using a custom-built protocol. The custom code for the Pipeline Pilot software (Accelrys) used to score T-cell and epithelial cell polarity will be provided upon request through a Materials Transfer Agreement (MTA) between the end-user institution and the Integrated Microscopy Core of Baylor College of Medicine.

The above protocol was modified slightly to process adherent epithelial cells, which were present at a higher density than Jurkat cells. Nuclear objects from the DAPI channel were detected using a local threshold with a window size of 60 × 60 pixels and C = 5. To identify overlapping nuclei, region splitting was applied to the objects by calculating an inverse distance transform on the thresholded image (that is, measuring a pixel’s distance to the nearest non-foreground pixel) and then finding peaks in the resulting distance map, where each peak corresponds to a nuclear seed. Starting from these seeds, a watershed transformation was run on the distance map to identify overlapping nuclei, which were discarded from the analysis using a shape filter. MTOC were detected as described for Jurkat cells. Leading edge cells were located visually from the DIC channel and then labeled manually with a red dot to mark the center of the leading edge. The centers of the nucleus, MTOC, and leading edge were calculated and used to determine the MTOC:leading edge angle.

### 3.5. Sorting MTOC:Bead Angles and MTOC:Leading Edge Angles into Designated Regions

Angles generated by the algorithm in Pipeline Pilot were sorted and analyzed in Microsoft Excel.

### 3.6. Statistics

ANOVA with Tukey post-hoc analysis using R statistical software was used.

## 4. Conclusions

This paper describes a new automated, high throughput system to score cell polarity establishment by capturing cell images, applying strict criteria to exclude unwanted cell images, compiling data, and calculating MTOC:bead angles. This flexible system permits sorting and evaluation of MTOC positions within cells using a variety of cell sectoring strategies. The system rapidly analyzes thousands of cells, facilitating acquisition of statistically significant results from very small regions of the cell and high resolution insight into the establishment of cell polarity. The approach has a variety of applications, including visualization and analysis of the virologic synapse and/or the role and effect of viral oncoproteins on polarity, and can be adapted for use in other systems and cell types. 
